# Splenic switch-off in three-dimensional adenosine stress cardiac magnetic resonance perfusion for differentiating true-negative from potentially false-negative studies identified by fractional flow reserve

**DOI:** 10.1016/j.jocmr.2025.101933

**Published:** 2025-07-17

**Authors:** Mihály Károlyi, Maximilian Fuetterer, Márton Kolossváry, Verena C. Wilzeck, Sven Plein, Andrea Biondo, Alexander Gotschy, Michael Frick, Rolf Gebker, Hatem Alkadhi, Ingo Paetsch, Cosima Jahnke, Sebastian Kozerke, Robert Manka

**Affiliations:** a Diagnostic and Interventional Radiology, University Hospital Zurich, University of Zurich, Zurich, Switzerland; b Department of Cardiology, University Heart Center, University Hospital Zurich, University of Zurich, Zurich, Switzerland; c Institute for Biomedical Engineering, University and ETH Zurich, Zurich, Switzerland; d Gottsegen National Cardiovascular Center, Budapest, Hungary; e Physiological Controls Research Center, University Research and Innovation Center, Óbuda University, Budapest, Hungary; f Multidisciplinary Cardiovascular Research Centre and the Department of Biomedical Imaging, Science, Leeds Institute of Cardiovascular and Metabolic Medicine, University of Leeds, Leeds, UK; g Department of Internal Medicine I, Cardiology, Angiology and Internal Intensive Care Medicine, University Hospital RWTH Aachen University, Aachen, Germany; h MVZ Diagnostikum Berlin 2020 GmbH, Berlin, Germany; i Department of Electrophysiology, HELIOS Heart Center Leipzig at University of Leipzig, Leipzig, Germany

**Keywords:** Coronary artery disease, Fractional flow reserve, Myocardial perfusion imaging, Cardiac magnetic resonance

## Abstract

**Background:**

False-negative cardiovascular magnetic resonance (CMR) perfusion results may arise from inadequate stress responses, even when patients exhibit an adequate clinical or heart-rate response to adenosine. This study aimed to explore the ability of qualitative and quantitative splenic switch-off (SSO) markers to differentiate true-negative from potentially false-negative adenosine stress-perfusion CMR findings in a cohort where fractional flow reserve (FFR) was used to adjudicate lesion significance.

**Methods:**

Patients with known or suspected coronary artery disease (CAD) from five centers underwent three-dimensional (3D) adenosine stress perfusion CMR and coronary angiography with FFR. SSO was assessed qualitatively using both standard stress-to-rest (SSO) and a stress-only (SSO_stress_) approach. In addition, quantitative signal intensity (SI) ratios were assessed, including the splenic stress-to-rest SI-ratio (SI_stress/rest_) and the spleen-to-myocardium SI ratio at stress (SI_spleen/myocarcium_). The diagnostic accuracy of these measures was evaluated using cross-validated area under the curve (cvAUC) analysis.

**Results:**

Among 179 patients (mean age 63 ± 10 years; 130 male), SSO prevalence was 73% (130/179) and was significantly more frequent in true-negative than false-negative CMR cases (80.6% [54/67] vs 36.8% [7/19], *p* < 0.001). SSO_stress_ showed moderate agreement (κ = 0.60) and robust diagnostic performance (AUC 0.80), as compared to SSO. Splenic SI_stress/rest_ and SI_spleen/myocarcium_ at stress demonstrated high predictive accuracy for visual SSO, with cvAUCs of 0.94 (95% CI: 0.90–0.96) and 0.90 (95% CI: 0.86–0.95), respectively. The positive likelihood ratio of SSO for true-negative CMR was 1.70, while the negative likelihood ratio was 0.24. Qualitative and quantitative splenic-switch off metrics classified 77%–80% (66-69/86) of negative CMR cases correctly as true- or potentially false-negatives, with sensitivities ranging from 81.4% to 91.2%. Clinically applicable cut-offs for differentiating true- and false-negative studies with splenic SI_stress/rest_ and SI_spleen/myocarcium_ at stress were identified as ≤0.32 and ≤0.38, respectively.

**Conclusion:**

In a multicenter cohort using FFR-adjudicated reference for lesion severity, qualitative SSO and quantitative SI metrics were associated with myocardial stress adequacy and these markers may improve the interpretation of negative stress-perfusion CMR studies.

## Graphical abstract

**Figure:**
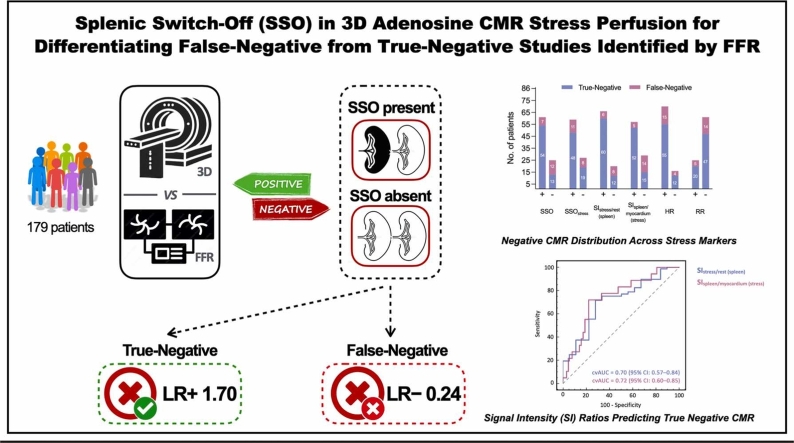

## 1 Background

Over the past decade, the use of non-invasive imaging for coronary artery disease (CAD) assessment has significantly expanded, with cardiovascular magnetic resonance (CMR) imaging, particularly with adenosine stress-perfusion protocols, emerging as a cornerstone for myocardial ischemia assessment [1]. Contemporary guidelines now provide Class I recommendations for functional imaging as the first diagnostic step in suspected CAD, reinforcing the pivotal role of CMR in clinical decision-making [2], [3], [4].

A critical aspect of adenosine stress CMR is ensuring an adequate vasodilatory response, as suboptimal stress may contribute to false-negative results. Conventional markers of pharmacologic stress adequacy—such as symptomatic responses and hemodynamic changes—are inconsistent predictors of adenosine-induced myocardial blood flow increase [5], [6]. To address this limitation, splenic switch-off (SSO) has been proposed as a physiological marker of adenosine-induced stress adequacy, reflecting the expected vasodilatory reduction in splanchnic perfusion during stress perfusion [7], [8].

The clinical value of SSO lies primarily in cases where stress CMR yields a negative result. If stress CMR is positive, patients will regularly undergo further evaluation with coronary angiography regardless of SSO findings. However, in negative cases, an inadequate stress response could lead to false-negative results, potentially delaying appropriate management. Therefore, the ability of SSO to distinguish true-negative from potentially false-negative studies is of particular clinical relevance.

Despite its potential, the clinical utility of SSO remains incompletely defined. Previous large-scale studies have primarily relied on qualitative visual assessment and defined SSO as the visual difference between the splenic signal intensity (SI) at stress compared to rest [7], [8]. However, in routine clinical practice, SSO is often evaluated solely on stress CMR images by visually comparing splenic SI to the myocardium during first-pass perfusion. Additionally, prior studies exploring the utility of SSO during CMR adenosine stress perfusion used quantitative coronary angiography (QCA) rather than fractional flow reserve (FFR) as the reference standard, limiting their ability to assess the physiological significance of coronary stenoses [7], [8].

This study addresses these gaps in current evidence by (1) evaluating the ability of qualitative SSO to differentiate true-negative from potentially false-negative CMR perfusion results using invasive FFR as the reference standard; (2) assessing the accuracy and clinical utility of a simplified, stress-only qualitative assessment (SSO_stress_); (3) testing quantitative splenic SI biomarkers to enable objective, reader-independent assessment of stress adequacy.

Unlike prior studies, we utilized three-dimensional (3D) CMR stress perfusion protocols that maximize splenic coverage, ensuring consistent splenic analysis across all cases. We hypothesized that both visual and quantitative SSO metrics could serve as reliable markers of myocardial stress adequacy during adenosine stress CMR perfusion, improving the interpretation of negative studies.

## 2 Methods

### 2.1 Study population

**Fig. 1:**
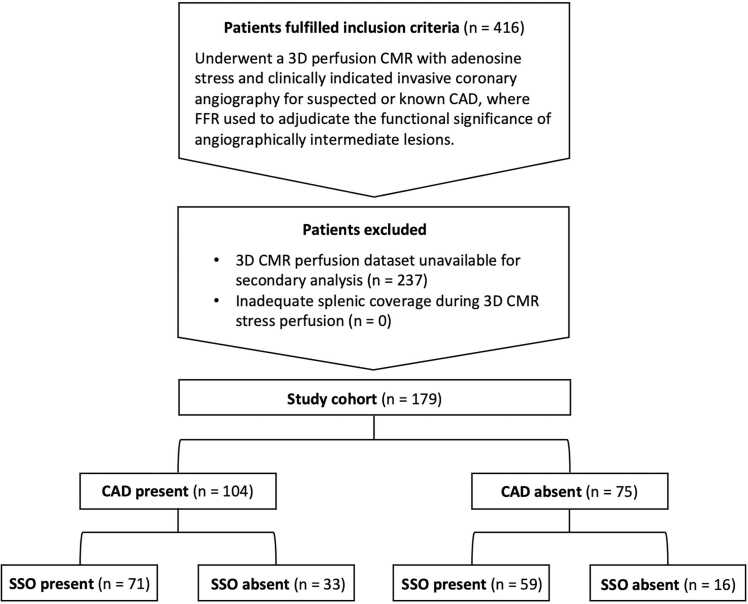
Patient inclusion flowchart showing initial enrollment, exclusions, and final study population. The number of patients with and without CAD (based on FFR ≤ 0.80) and their respective SSO status (present or absent) are also presented. *CAD* coronary artery disease, *FFR* fractional flow reserve, *SSO* splenic switch-off, *3D* three-dimensional, *CMR* cardiovascular magnetic resonance

This study retrospectively analyzed participants from a previously published multicenter study [9], [10], conducted between 2009 and 2013 at five centers. This post-hoc analysis comprised all patients from the original study. All participants underwent a 3D perfusion CMR with adenosine stress in addition to a clinically indicated invasive coronary angiography for suspected or known CAD, where FFR was used to adjudicate the functional significance of angiographically intermediate lesions. Contraindications for adenosine-perfusion CMR included bronchial asthma, high-degree atrioventricular block, metallic foreign bodies, or claustrophobia. Exclusion criteria for the current study included non-retrievable or incomplete 3D CMR datasets or insufficient splenic coverage on stress or rest imaging, preventing qualitative assessment of splenic perfusion or quantitative analysis due to the inability to place a region of interest (ROI) of at least 1 cm². Study protocol was approved by the local ethics committee in each participating center, and all patients provided written informed consent. As this was a post-hoc analysis of a predefined cohort, no separate a priori power calculation was performed; the sample size was determined by the number of eligible patients with complete imaging and invasive data. The study population is outlined in Fig. 1.

### 2.2 Myocardial perfusion CMR imaging

CMR examinations were performed at either 1.5T or 3T magnetic resonance imaging (MRI) systems using vector electrocardiogram (ECG) synchronization and multichannel torso coil arrays (5–32 channels). Imaging included cine sequences, whole-heart 3D first-pass perfusion sequences, and late gadolinium enhancement images. This analysis focused exclusively on 3D first-pass perfusion sequences, obtained at stress and rest, acquired in a short-axis orientation covering the entire left ventricle. Perfusion imaging used a saturation-recovery gradient-echo sequence, as previously described in the 3D perfusion imaging protocol [11]. Participants abstained from caffeine 24 h prior to imaging. Adenosine was administered intravenously with a standard dose of 140 µg/kg/min for at least 3 min to induce stress. A gadolinium-based contrast agent (0.1 mmol/kg) was injected, followed by a saline bolus. Rest and stress imaging were performed in a breath-hold with shallow expiration as needed, using identical acquisition protocols. Hemodynamic parameters, including heart rate (HR) and blood pressure (RR), were recorded during adenosine stress and at rest to monitor physiological responses to stress induction and ensure patient safety during the procedure.

### 2.3 Assessment of myocardial ischemia with perfusion CMR and invasive FFR

Perfusion CMR data were centrally analyzed for ischemia by experienced readers blinded to clinical and angiographic information. Myocardial ischemia was defined visually as a stress perfusion deficit with ≥25% transmurality persisting across ≥3 consecutive dynamics, without a corresponding rest perfusion defect or late gadolinium enhancement. For this study, ischemia was considered on a per-patient basis, classifying the entire CMR study as either positive or negative. This approach aimed to capture the overall myocardial response to adenosine, identifying ischemia in any myocardial segment to differentiate patients with CAD from those without.

Invasive coronary angiography was performed following standard protocols, with QCA conducted offline in a blinded core laboratory. FFR was selectively performed in vessels showing 50%–80% luminal stenosis in two orthogonal views and a diameter ≥2 mm. FFR <0.8 was deemed functionally significant, while stenosis >80% was considered hemodynamically significant without requiring FFR testing. Similar to CMR ischemia evaluation, for the present post-hoc analysis, FFR results were classified on a per-patient basis, ensuring that ischemia was accounted for if present in any vessel. This approach aligns with the strategy of evaluating myocardial ischemia as a whole-organ response.

### 2.4 Assessment of SSO

Qualitative SSO and simple quantitative SI ratios were analyzed using commercially available software (OsiriX MD, version 12.0, Geneva, Switzerland). A single experienced observer (M.K., Level 3 certification) conducted image analysis while blinded to clinical data. Qualitative SSO was visually assessed by comparing splenic enhancement between stress and rest CMR images, focusing on maximal splenic visibility. SSO was defined as a visual reduction in splenic SI during stress, observed at the time of maximum myocardial SI during first-pass perfusion, compared to rest [8]. Additionally, a simplified qualitative assessment technique was evaluated, in which splenic enhancement was visually judged relative to the myocardium on stress images alone (SSO_stress_), at peak myocardial first-pass perfusion. SSO_stress_ assessment was performed by the same reader (M.K.), blinded to clinical data and the original SSO assessment, 8 weeks after the initial reading. Failed visual SSO was defined as either similar splenic enhancement at both stress and rest or splenic enhancement comparable to that of the myocardium. Simple quantitative ratios were assessed at the time of peak myocardial SI during first-pass perfusion. First, ROIs were placed on consecutive time frames of the perfusion scan in a remote myocardial segment without corresponding ischemia or late gadolinium enhancement, and the time frame with the highest SI was selected. Second, on the selected time frame, the slice with the largest splenic coverage within the 3D dataset was chosen, and regions of interests (ROIs) were placed on the spleen and remote myocardium. These ROIs were copied between stress and rest images using the software’s copy-paste function. The stress-to-rest SI ratio (SI_stress/rest_) of the spleen was calculated as: (SI_spleen stress_−SI_spleen pre-contrast_) / (SI_spleen rest_−SI_spleen pre-contrast_). Similarly, the splenic-to-myocardial SI ratio (SI_spleen/myocardium_) at stress was determined as: (SI_spleen stress_−SI_spleen pre-contrast_) / (SI_myocardium stress_−SI_myocardium pre-contrast_)_._ SI values were normalized by subtracting baseline (pre-contrast) SI measurements. All SI values were measured in arbitrary units (a.u.).

**Fig. 2:**
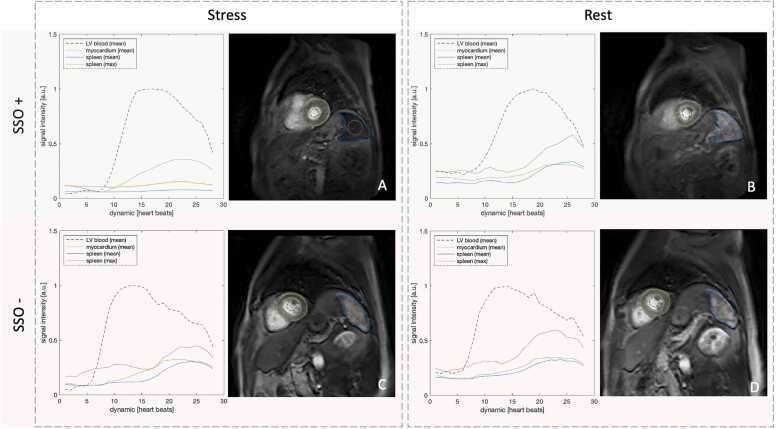
Example of splenic switch-off (SSO) and signal intensity (SI) assessment. Representative first-pass perfusion images at peak myocardial signal intensity from a patient with splenic switch-off (SSO+) during 3D adenosine stress perfusion (A), and the corresponding rest perfusion image (B). Images (C) and (D) show another patient with absent splenic switch-off (SSO−) during adenosine stress and rest, respectively. Corresponding SI-time curves display: splenic mean (blue solid line) and maximum (orange solid line) SI, left ventricular (LV) blood pool mean SI (purple dotted line), and myocardial mean SI (green solid line). SI values are normalized to the peak LV blood pool signal and are given in arbitrary units (a.u.).While the anterolateral wall in panel C appears relatively hypointense, the patient had no perfusion defect on clinical read and no flow-limiting lesion on FFR. Furthermore, the green SI curve demonstrates similar myocardial signal enhancement between stress and rest, indicating no quantitative evidence of ischemia. *FFR* fractional flow reserve.

Advanced signal post-processing and perfusion quantification were performed using the Agora research platform (Gyrotools LLC, Zurich, Switzerland) and MATLAB (MathWorks, Natick, Massachusetts, version 2023b) in order to identify further quantitative biomarkers to assess SSO. For this analysis, ROIs were placed on the slices where the spleen was most visible within the 3D dataset and copied across all frames of the first-pass perfusion sequence. Parameters such as time-to-peak (TTP) SI and upslope during stress (defined as the change between minimum and maximum SI) were extracted. Inter-observer reliability was assessed in a randomly selected subset comprising 25% of the cohort for each technique (V.C.W., Level 3 certification), and intra-observer readings were performed after an 8-week interval (M.K.). Examples of patients with present and failed SSO from the study cohort are shown in Fig. 2.

### 2.5 Classification of negative CMR findings based on SSO

Negative CMR cases (no stress perfusion deficits) were analyzed to assess SSO’s role in differentiating true-negative from potentially false-negative cases. SSO presence and absence were compared with FFR-based true- and false-negative classifications, respectively. Positive and negative likelihood ratios were calculated to assess the ability of SSO to differentiate between FFR-based true-negative and false-negative cases among negative CMR studies. Similarly, thresholds of SI_stress/rest_ of the spleen and SI_spleen/myocardium_ at stress were identified and tested for the differentiation of true-negative from potentially false-negative results.

### 2.6 Statistical analysis

Statistical analyses were performed using SPSS (version 23; IBM Corp., Armonk, New York) and R (version 3.6.1; R Foundation for Statistical Computing, Vienna, Austria; https://www.r-project.org). Continuous data are reported as mean ± standard deviation and analyzed using Student’s *t*-test or Mann-Whitney *U* test, as appropriate. Categorical data were analyzed using the Chi-squared test. Agreement between visual and simplified stress-only SSO methods was evaluated using Cohen’s Kappa and receiver operating characteristic (ROC) analysis. Diagnostic performance of quantitative SSO parameters was evaluated using area under the curve (AUC) from ROC curves with 5-fold cross-validation (cvAUC package ver. 1.1.4 in R), and Youden’s J statistic determined optimal cutoffs. Reliability metrics were assessed with intraclass correlation coefficients (ICC), categorized as poor (<0.5), moderate (0.5–<0.75), good (0.75–0.9), or excellent (≥0.9) [12]. Statistical significance was set at a 2-sided *p* < 0.05.

## 3 Results

### 3.1 Patient demographics, CMR parameters, and diagnostic findings

From the original cohort of 416 patients, 179 patients were included into this post-hoc analysis due to data availability constraints. Missing images were entirely random, with no patient characteristics associated with data unavailability, as shown in Supplementary Table. Adequate splenic coverage was available in all included cases, and no patients were excluded. The study population and patient stratification based on the presence or absence of CAD (defined by FFR) and corresponding SSO status are outlined in Fig. 1. Baseline parameters and diagnostic findings are summarized in Table 1, Table 2, Table 3, respectively.

**Table 1:** Clinical characteristics of study population

	*n = 179*
*Baseline characteristics*	
Age (years)	63 ± 10
Male, *n* (%)	130 (73)
BMI (kg/m^2^), mean ± SD	28 ± 4
*Cardiovascular risk factors*	
Hypertension, *n* (%)	130 (73)
Diabetes, *n* (%)	31 (17)
Dyslipidemia, *n* (%)	116 (65)
Smoker, *n* (%)	74 (41)
Family risk of CAD, *n* (%)	61 (34)
*Regular medication*	
ACE-inhibitor, *n* (%)	84 (47)
ARB, *n* (%)	30 (17)
Beta-blocker, *n* (%)	92 (51)
Calcium-antagonist, *n* (%)	34 (19)
Diuretic, *n* (%)	33 (18)
Nitrate, *n* (%)	14 (8)
Statin, *n* (%)	114 (64)

*ACE* angiotensin-converting, *ARB* angiotensin receptor blocker, *BMI* body mass index, *CAD* coronary artery disease, *SD* standard deviation

**Table 2:** Invasive coronary angiography diagnostic findings

	*n* = 179
CAD (>50% luminal stenosis), *n* (%)	114 (64)
Single-vessel disease, *n* (%)	53 (30)
Multi-vessel disease, *n* (%)	61 (34)
Pathological FFR (<0.8), *n* (%)	104 (58)

*CAD* coronary artery disease; *FFR* fractional flow reserve

**Table 3:** CMR hemodynamic parameters and diagnostic findings

	*n* = 179
*CMR perfusion parameters*	
*Stress:*	
Heart-rate (bpm), mean ± SD	82 ± 15
Systolic blood-pressure (mmHg), mean ± SD	127 ± 21
Diastolic blood-pressure (mmHg), mean ± SD	71 ± 10
*Rest:*	
Heart-rate (bpm), mean ± SD	66 ± 12
Systolic blood-pressure (mmHg), mean ± SD	129 ± 21
Diastolic blood-pressure (mmHg), mean ± SD	73 ± 10
*CMR stress perfusion diagnostic findings (FFR as reference)*	
Positive, *n* (%)	93 (52)
True positive, *n* (%)	85 (47)
False positive, *n* (%)	8 (5)
Negative, *n* (%)	86 (48)
True negative, *n* (%)	67 (37)
False negative, *n* (%)	19 (11)

*CMR* cardiovascular magnetic resonance; *FFR* fractional flow reserve, *SD* standard deviation.

### 3.2 SSO as an indicator of true negative CMR results

**Fig. 3:**
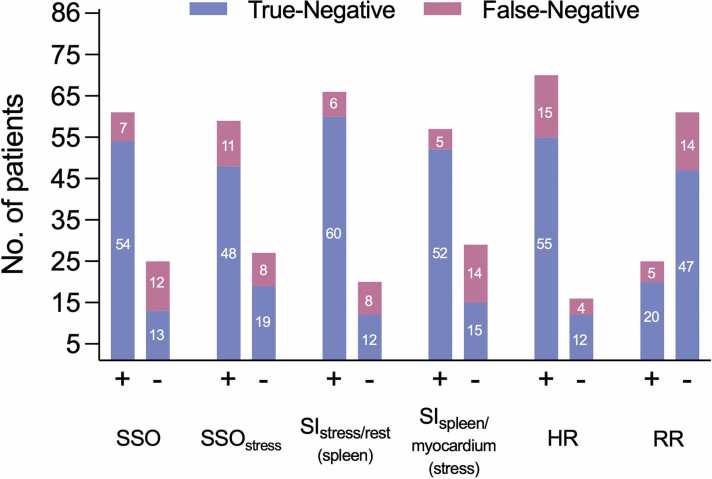
Markers of stress adequacy in CMR. This diagram depicts the distribution of true-negative and false-negative CMR perfusion results among negative CMR studies, stratified by the presence or absence of different stress adequacy markers, with invasive FFR serving as the reference standard. The following thresholds were used to define an adequate stress response: SI ratio stress-to-rest of spleen ≤0.32, SI ratio spleen-to-myocardium at stress ≤0.38, heart rate increase ≥10 bpm, and blood pressure decrease ≥10 mmHg. *CMR* cardiac magnetic resonance, *FFR* fractional flow reserve, *HR* heart rate, *RR* blood pressure, *SI* signal, *SSO* splenic switch-off between stress and rest

Visual SSO was present in 73% (130/179) of the cohort, with a significantly higher prevalence among true-negative versus false-negative CMR studies (80.6% [54/67] vs 36.8% [7/19], *p* < 0.001). Among all negative CMR scans (*n* = 86), visual SSO was observed in 78% (61/86), correctly identifying 63% (54/86) as true-negative. In contrast, absence of SSO was coincided with a higher false-negative rate (14%, 12/86). Fig. 3 illustrates this distribution.

Overall, 77% (66/86) of negative CMR studies were consistent with true- or false negative classification based on visual SSO, yielding a positive likelihood ratio of 1.70 and a negative likelihood ratio of 0.24 (Table 4). Inter- and intra-observer reliability for SSO were good, with ICCs of 0.82 (95% CI: 0.66–0.90) and 0.87 (95% CI: 0.73–0.93), respectively.

**Table 4:** Predictive performance of stress adequacy indicators for identifying true-negative CMR diagnoses (vs FFR)

	SSO	SSO_stress_	SI_stress/rest__(spleen)_	SI_spleen/myocardium__(stress)_	HR	RR
Sensitivity (%)	88.5	81.4	91.2	91.2	78.6	80.0
Specificity (%)	48.0	29.6	41.2	48.3	25.0	23.0
Accuracy (%)	76.7	65.1	74.3	76.7	59.3	39.5
LR+	1.70	1.16	1.55	1.76	1.05	1.03
LR−	0.24	0.63	0.21	0.18	0.85	0.87

This table summarizes the predictive performance of stress adequacy markers for classifying negative CMR perfusion studies as true-negative or potentially false-negative, using FFR as the reference standard for CAD. The metrics were derived within the subgroup of patients with negative stress perfusion imaging. Higher LR+ values indicate a greater likelihood of a true-negative classification when the marker (e.g. SSO) is present; conversely, lower LR− values suggest that absence of the marker decreases the likelihood of a true-negative result, thereby raising suspicion for a potential false-negative CMR. Thresholds for stress adequacy were SI_stress/rest_ of spleen ≤ 0.32, SI_spleen/myocardium_ at rest ≤0.38, HR increase ≥10 bpm, and RR decrease ≥10 mmHg

*LR+* positive likelihood ratio, *LR*− negative likelihood ratio, *SSO* splenic switch-off between stress and rest, *SSO_stress_* splenic switch-off at stress only, *SI_stress/rest_*_*(spleen)*_ splenic stress-to-rest ratio, *SI_spleen/myocardium_*_*(stress)*_ splenic-to-myocardial ratio at stress, *CMR* cardiovascular magnetic resonance, *FFR* fractional flow reserve, *HR* heart rate response, *RR* blood pressure response.

### 3.3 Simplified qualitative SSO assessment using stress-only CMR images

SSO_stress_ demonstrated moderate agreement with the original stress-rest method (Cohen’s kappa: 0.60, 95% CI: 0.59–0.85). Sensitivity and specificity for the simplified method were 72.6% and 70.0%, compared to SSO, respectively. ROC analysis showed good alignment between the two techniques, with an AUC of 0.80 (95% CI: 0.72–0.88). Intra- and inter-observer reliability for visual SSO_stress_ was good, with ICCs of 0.84 (95% CI: 0.70–0.91) and 0.78 (95% CI: 0.62–0.88), respectively. Diagnostic performance of SSO_stress_ in summarized in Table 4.

### 3.4 Quantitative biomarkers predicting visual SSO

Quantitative splenic perfusion parameters demonstrated high predictive accuracy for SSO, with the SI_stress/rest_ of the spleen yielding a cvAUC of 0.94 (95% CI: 0.90–0.96), while SI_spleen/myocardium_ at stress showed a cvAUC of 0.90 (95% CI: 0.86–0.95). Optimal thresholds for SSO-prediction were identified as ≤0.25 for the splenic SI_stress/rest_ (sensitivity: 93.2%, specificity: 80.8%) and ≤0.28 for the SI_spleen/myocardium_ at stress (sensitivity: 91.8%, specificity: 86.9%). Other assessed parameters, including upslope and TTP during stress, demonstrated poor diagnostic performance, with cvAUCs of 0.46 (95% CI: 0.35–0.57) and 0.44 (95% CI: 0.34–0.55), respectively. Intra- and inter-observer reliability for SI ratios between stress and rest imaging were good, with ICCs of 0.85 (95% CI: 0.70–0.92) and 0.83 (95% CI: 0.68–0.91).

### 3.5 Quantitative biomarkers for predicting true-negative stress perfusion

**Fig. 4:**
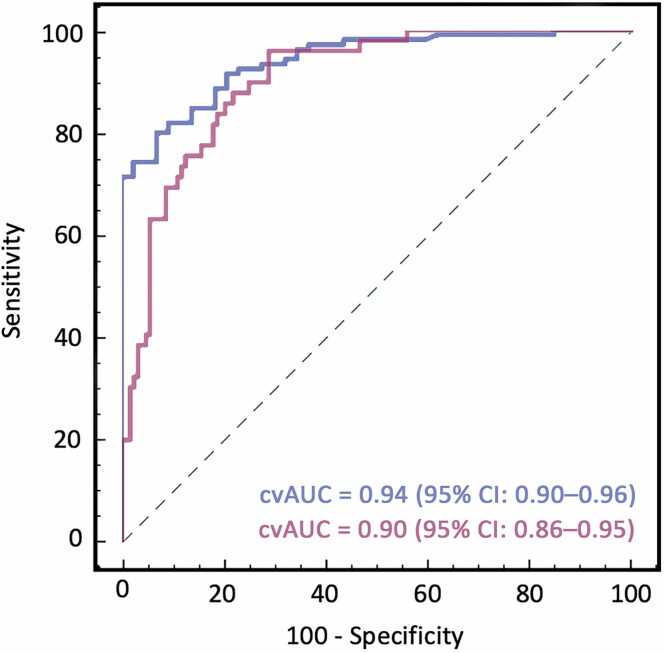
Predictive performance of signal intensity ratios for visual splenic switch-off. Receiver operating characteristic (ROC) curves demonstrating the ability of quantitative splenic signal intensity ratios to predict visually assessed splenic switch-off (SSO). Both the stress-to-rest signal intensity ratio of the spleen (blue) and the splenic-to-myocardial signal intensity ratio under stress (purple) showed excellent agreement with visual SSO status. These findings support the use of objective markers to complement or potentially replace visual evaluation in the absence of rest images. *cvAUC* cross-validated area under the curve, *CMR* cardiovascular magnetic resonance, *FFR* fractional flow reserve

**Fig. 5:**
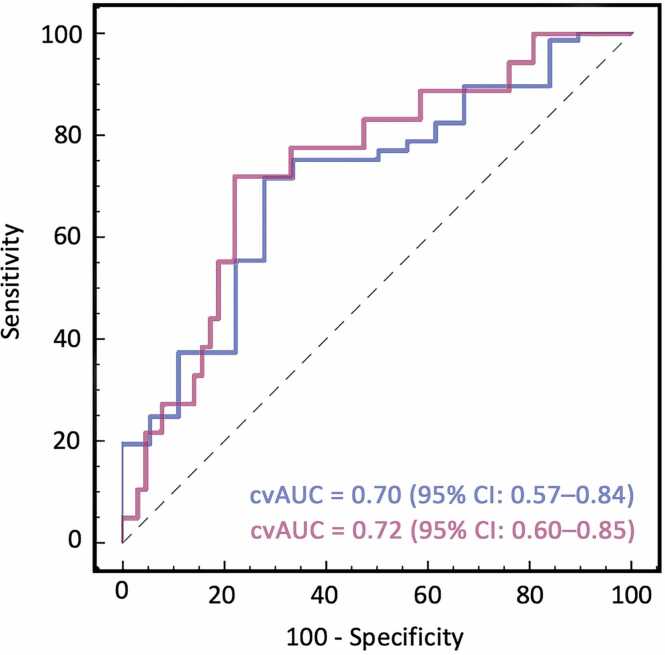
Ability of signal intensity ratios for identifying true-negative CMR studies. ROC curves for evaluating the ability of quantitative splenic perfusion parameters to discriminate between true-negative and potentially false-negative CMR perfusion studies (using invasive FFR as the reference standard). Both the stress-to-rest signal intensity ratio of the spleen (blue) and the splenic-to-myocardial ratio at stress (purple) showed good predictive ability in identifying true-negative studies, indicating their potential role as objective markers of stress adequacy among negative CMR exams. *cvAUC* cross-validated area under the curve, *CMR* cardiovascular magnetic resonance, *FFR* fractional flow reserve, *ROC* receiver operating characteristic

Among negative CMR studies, splenic SI_stress/rest_ demonstrated moderate accuracy for true-negative classification, with a cvAUC of 0.70 (95% CI: 0.57–0.84). At a threshold of ≤0.32, it correctly classified 69.7% (60/86) as true-negative and identified 10.4% (9/86) as potentially false-negative. The overall classification accuracy was 80.2% (69/86). SI_spleen/myocardium_ at stress achieved a cvAUC of 0.72 (95% CI: 0.60–0.85), with a threshold of ≤0.38 correctly identifying 60% (52/86) as true-negative and 16% (14/86) as potentially false-negative. In contrast, upslope and TTP showed limited predictive value, with cvAUCs of 0.42 (95% CI: 0.26–0.58) and 0.46 (95% CI: 0.29–0.62). Predictive performance of quantitative parameters for SSO is illustrated in Fig. 4. Fig. 5 demonstrates cvAUC curves for splenic SI ratios in predicting true-negative CMR studies.

## 4 Discussion

This post-hoc analysis of a multicenter cohort reinforces the role of SSO as a robust marker of myocardial stress adequacy during adenosine stress perfusion CMR. In this multicenter study, which used FFR to determine the functional relevance of angiographically intermediate lesions, SSO was associated with true-negative CMR results, supporting its potential as a diagnostic adjunct. Additionally, we identified quantitative splenic SI biomarkers that offer standardized assessment of stress adequacy. A simplified, stress-only SSO assessment technique demonstrated moderate agreement with conventional stress-rest comparisons in our study, which may enhance clinical workflow efficiency. Notably, spleen was assessable in 100% of our cohort due to the expanded 3D coverage of the applied perfusion sequence. In contrast, previous studies reported splenic visibility rates of 96%–99% [8], [13], justifying the suitability of this imaging approach for our study objectives.

SSO reflects a reduction in splenic perfusion during adenosine stress, which is attributed to adenosine-induced vasoconstriction within the splanchnic circulation, leading to a redistribution of blood flow away from the splanchnic circulation, including the spleen. Prior studies using Doppler ultrasound and nuclear techniques have reported reductions in splanchnic organ perfusion during pharmacologic stress, supporting this mechanism [13], [14]. While SSO is not a direct measure of splanchnic flow, the visibly decreased splenic SI on stress perfusion images in CMR images reflects this physiological response. The role of SSO as a marker of myocardial stress adequacy in adenosine perfusion CMR was first highlighted by Manisty et al. in 2015 within the Clinical Evaluation of Magnetic Resonance Imaging in Coronary Heart Disease (CE-MARC) cohort, using QCA as the reference standard [8]. Subsequent studies have further elucidated its clinical utility in real-world cohorts and explored variations in SSO prevalence across specific patient subgroups [15], [16], [17]. Our work builds on this foundation by evaluating the performance of SSO in a cohort where lesion significance was defined by invasive FFR.

The prevalence of SSO in our cohort (73%) was similar to that reported in a previous study (72%), where adenosine was administered at the standard dose of 140 µg/kg/min, as in our study [13]. In studies using higher adenosine doses (140–210 µg/kg/min), SSO was observed in up to 89%–93% of cases [7], [8], [18]. While the lower prevalence in our cohort may reflect a relatively lower stress burden, we still confirmed that SSO was significantly more frequent in true-negative than in false-negative cases, emphasizing its role in confirming adequate myocardial stress. A higher adenosine dose might have further strengthened our findings.

We identified SI_stress/rest_ of the spleen as a highly accurate predictor of qualitative SSO, with a cross-validated AUC of 0.94 and an optimal cut-off of ≤0.25 (sensitivity: 93.2%, specificity: 80.8%). This closely aligns with Hosking et al., who reported an AUC of 0.91 (sensitivity: 82.5%, specificity: 92.3%), though their optimal splenic SI_stress/rest_ was slightly higher (0.40) [7]. A more recent study by Patriki et al., using hybrid ¹³N-ammonia PET and 3T CMR during adenosine-induced stress reported even higher ratio of 0.71 for SSO prediction (sensitivity: 94%, specificity: 94%, AUC = 0.947) [13]. While the authors attributed this discrepancy more likely to different adenosine dosing, the difference to our result may stem from different splenic SI_stress/rest_ definitions: while we assessed splenic stress and rest signal intensities at the time of maximal myocardial first-pass perfusion, the other two studies may have compared maximal splenic signal intensities across stress and rest [7], [13].

Importantly, as splenic-switch-off can be assessed on stress-only images alone, it theoretically enabling real-time evaluation of stress adequacy during routine CMR. Its primary advantage lies in its alignment with current clinical protocols, as most institutions now omit rest perfusion imaging to streamline workflows and reduce scan duration [19], in accordance with guidelines advocating for stress-only protocols whenever feasible [6]. This is especially relevant as the number of non-invasive imaging studies for CAD continues to grow [1], driving efforts to improve patient throughput and comfort. Advances in acceleration techniques, including those enabled by deep learning, further facilitate shorter acquisition protocols. In this evolving context, stress-only SSO assessment may offer a practical adjunct for real-time evaluation of vasodilator response [20]. Building on this, beyond the original definition of visual SSO, which compares splenic perfusion between stress and rest, our study also evaluated stress-only approaches for SSO assessment. A simplified qualitative method, SSO_stress_ demonstrated moderate agreement with the conventional stress-rest method (Cohen’s kappa: 0.60) in our study. While slightly less sensitive and specific, SSO_stress_ correlated well with SSO and represents a clinically viable alternative. Incorporating SSO_stress_ into CMR workflows could enhance efficiency, particularly in high-volume settings. Similarly, SI_spleen/myocardium_ at stress, a quantitative metric of SSO derived from stress-only images, demonstrated high accuracy in predicting qualitative SSO, with a cross-validated AUC of 0.90 (95% CI: 0.86–0.95) and an optimal cut-off of ≤0.28, achieving a sensitivity of 91.8% and a specificity of 86.9%. However, stress-only assessment may also have limitations. The absence of rest imaging could reduce diagnostic certainty in borderline cases, and visual interpretation may be less reliable in low-contrast situations. Moreover, although our results stem from a multicenter cohort, broader validation across institutions and scanner platforms is warranted before wide adoption. Interestingly, Patriki et al. reported a markedly different SI_spleen/myocardium_ threshold at stress to predict visual SSO (1.53, sensitivity: 61%, specificity: 85%, AUC = 0.76) [13]. However, this threshold appears counterintuitive, as a splenic-to-myocardial SI ratio above 1 contradicts the expected perfusion pattern in SSO, where splenic SI should be lower than myocardial SI [13]. Nevertheless, similar to SI_spleen/myocardium_ at stress, the primary reason for the discrepancies of their finding and ours likely lies in differences in perfusion metric definitions. While Patriki et al. may have derived maximal splenic and myocardial SIs at stress from perfusion curves, potentially from different time frames [13], we assessed SI ratios at the time of maximal myocardial SI during first-pass perfusion, ensuring both values were derived from the same time frame. This approach is more practical for routine clinical use, as it eliminates the need for perfusion curve analysis. Other contributing factors may include variations in imaging modalities, scanner settings, perfusion protocols (e.g., stress-rest order and timing), and patient populations.

In our study, quantitative splenic perfusion ratios deemed also valuable for predicting a true-negative CMR study. Splenic SI_stress/rest_ classified negative CMR cases with an accuracy of 80.2% as either true- or potentially false negative, while SI_spleen/myocardium_ at stress demonstrated comparable performance. Our findings suggest that these metrics may provide practical cut-offs to aid the clinical interpretation of negative CMR studies. SI_stress/rest_ of the spleen, in particular, may offer several potential advantages over conventional visual SSO assessment, including increased objectivity, reduced inter-observer variability, and the potential for automation and standardization of stress adequacy evaluation. It may also serve as a helpful adjunct in borderline or ambiguous cases where visual SSO is inconclusive. However, similar to the thresholds for predicting SSO, these cut-offs may vary depending on the study cohort, imaging technique used, and biomarker definitions. Moreover, the use of the stress-to-rest SI ratio of the spleen requires both stress and rest perfusion images, which may limit its utility in centers using stress-only protocols. Therefore, relying solely on absolute thresholds for SSO assessment needs further validation in larger, diverse cohorts to establish universally applicable cut-off values. Advanced quantitative parameters, such as TTP and upslope had limited predictive value in our study, emphasizing the need for further refinement of myocardial and splenic perfusion quantification techniques.

## 5 Limitations

Despite its insights, our study has limitations. While FFR was not used to assess stress adequacy directly, it served to adjudicate the significance of intermediate lesions, allowing classification of CMR results as true- or potentially false-negative. Importantly, no outcome data (e.g., major adverse cardiovascular events) were available for this post-hoc analysis, as follow-up was not part of the original study design. Although prognostic data would offer valuable complementary insights, the primary aim was to evaluate diagnostic performance and assess SSO as a marker of stress adequacy. Therefore, the lack of follow-up does not compromise the study objectives. Although we cross-validated our results to provide a better unbiased estimate of real-world performance, as a post-hoc analysis, the identified SSO prevalence and quantitative cut-offs may reflect the specific CAD prevalence and imaging protocols of this cohort. Also, the use of a standardized 140 µg/kg/min adenosine dose may have influenced SSO specificity. Previous studies suggest higher specificity with increasing doses [18], warranting future investigations into optimized adenosine titration strategies. Additionally, while we did not specifically analyze atrial fibrillation as a confounder, prior studies suggest that SSO is less prevalent in patients with atrial fibrillation compared to those in sinus rhythm [15]. This may impact the generalizability of our findings, particularly in populations with a high prevalence of atrial fibrillation. While derived from data between 2009 and 2013, our findings of visual SSO and simple ratios likely remain applicable with modern CMR technology, though advanced acquisition and reconstruction techniques might enhance results for parameters like upslope and TTP, possibly enabling simultaneous quantification of myocardial and splenic blood flow. Advances in imaging, such as T1 mapping and quantitative perfusion techniques, may further enhance the evaluation of myocardial and splenic stress adequacy, enabling simultaneous measurement of myocardial and splenic blood flow [21]. Lastly, SSO does not occur with alternative vasodilators such as regadenoson, restricting the applicability of our results to adenosine-based stress CMR protocols.

## 6 Conclusion

In conclusion, this study provides evidence supporting the use of SSO as an indicator of myocardial stress in adenosine stress-perfusion CMR. Quantitative splenic perfusion metrics provide objective tools for assessing splenic switch-off, which may enhance the reproducibility and efficiency of CMR interpretation. Furthermore, the simplified stress-only SSO assessment appears promising for clinical implementation, underscoring the potential value of SSO-based evaluation in contemporary CMR workflows.

## Author contributions

**Mihály Károlyi:** Writing – review & editing, Writing – original draft, Visualization, Validation, Supervision, Project administration, Methodology, Investigation, Formal analysis, Data curation, Conceptualization. **Maximilian Fuetterer:** Validation, Methodology, Formal analysis, Conceptualization. **Márton Kolossváry:** Writing – review & editing, Methodology, Formal analysis. **Verena C. Wilzeck:** Validation, Investigation, Formal analysis. **Sven Plein:** Validation, Supervision, Methodology, Conceptualization. **Andrea Biondo:** Methodology, Investigation, Formal analysis. **Alexander Gotschy:** Validation, Supervision, Conceptualization. **Michael Frick:** Visualization, Validation, Resources, Methodology. **Rolf Gebker:** Validation, Supervision, Resources. **Hatem Alkadhi:** Writing – review & editing, Supervision, Investigation. **Ingo Paetsch:** Writing – review & editing, Supervision, Methodology, Conceptualization. **Cosima Jahnke:** Writing – review & editing, Validation, Supervision. **Sebastian Kozerke:** Writing – review & editing, Visualization, Validation, Project administration. **Robert Manka:** Writing – review & editing, Visualization, Validation, Supervision, Resources, Conceptualization.

## Declaration of competing interests

The authors declare the following financial interests/personal relationships, which may be considered as potential competing interests. Mihaly Karolyi, Verena C. Wilzeck, Andrea Biondo, Alexander Gotschy, Hatem Alkadhi, and Robert Manka report a relationship with Bayer AG that includes funding grants. Mihaly Karolyi, Verena C. Wilzeck, Andrea Biondo, Alexander Gotschy, Hatem Alkadhi, and Robert Manka report a relationship with Canon Medical Systems Corporation that includes funding grants. Mihaly Karolyi, Verena C. Wilzeck, Andrea Biondo, Alexander Gotschy, Hatem Alkadhi, and Robert Manka report a relationship with Guerbet that includes funding grants. Mihaly Karolyi, Verena C. Wilzeck, Andrea Biondo, Alexander Gotschy, Hatem Alkadhi, and Robert Manka report a relationship with Siemens AG that includes funding grants. Robert Manka reports a relationship with Swiss Heart Foundation that includes funding grants. Robert Manka reports a relationship with University Hospital Zurich that includes funding grants. Robert Manka reports a relationship with Philips that includes speaking and lecture fees. Robert Manka reports a relationship with Bristol Myers Squibb that includes speaking and lecture fees. Hatem Alkadhi and Robert Manka report a relationship with Siemens AG that includes speaking and lecture fees. The other authors declare that they have no known competing financial interests or personal relationships that could have appeared to influence the work reported in this paper.
