# Supplementary material for: Splenic switch-off in three-dimensional adenosine stress cardiac magnetic resonance perfusion for differentiating true-negative from potentially false-negative studies identified by fractional flow reserve
Source: J Cardiovasc Magn Reson. 2025 Jul 17;27(2):101933. doi: 10.1016/j.jocmr.2025.101933 (PMC12670903; doi:10.1016/j.jocmr.2025.101933)
Supplement: Supplementary file 1 — Supplementary material [file mmc1.docx]

**Supplementary Table** Baseline characteristics and imaging data of study population and excluded patients.

|  | *Study population* | *Excluded patients* |  |
| --- | --- | --- | --- |
|  | *n=179* | *n=237* | *p-value* |
| **Baseline characteristics** |  |  |  |
| Age (years) | 63 ± 10 | 63 ± 11 | 0.54 |
| Male, n (%) | 130 (73) | 192 (81) | 0.63 |
| BMI (kg/m^2^), mean ± SD | 28 ± 4 | 27 ± 4 | 0.12 |
| **Cardiovascular risk factors** |  |  |  |
| Hypertension, n (%) | 130 (73) | 188 (79) | 0.11 |
| Diabetes, n (%) | 31 (17) | 59 (25) | 0.06 |
| Dyslipidemia, n (%) | 116 (65) | 157 (66) | 0.71 |
| Smoker, n (%) | 66 (37) | 75 (32) | 0.27 |
| Family risk of CAD, n (%) | 61 (34) | 52 (22) | <0.01 |
| **Hemodynamic parameters during CMR stress** |  |  |  |
| Baseline HR (bpm), mean ± SD | 67 ± 12 | 67 ± 10 | 0.49 |
| Maximum HR (bpm), mean ± SD | 84 ± 15 | 83 ± 14 | 0.26 |
| Baseline RRsyst (mmHg), mean ± SD | 129 ± 21 | 130 ± 19 | 0.50 |
| Maximum RRsyst (mmHg), mean ± SD | 127 ± 20 | 130 ± 21 | 0.29 |
| Baseline RRdiast (mmHg), mean ± SD | 72 ± 10 | 72 ± 11 | 0.06 |
| Maximum RRdiast (mmHg), mean ± SD | 71 ± 10 | 70 ± 10 | 0.50 |
| **Imaging findings** |  |  |  |
| CMR stress test positive, n (%) | 93 (52) | 141 (59) | 0.13 |
| Significant CAD (FFR) | 104 (58) | 120 (51) | 0.13 |

BMI body mass index, CAD coronary artery disease, HR heart rate, RR Blood pressure, SD standard deviation.
